# Investigation of a Genome Wide Association Signal for Obesity: Synthetic Association and Haplotype Analyses at the Melanocortin 4 Receptor Gene Locus

**DOI:** 10.1371/journal.pone.0013967

**Published:** 2010-11-15

**Authors:** André Scherag, Ivonne Jarick, Jessica Grothe, Heike Biebermann, Susann Scherag, Anna-Lena Volckmar, Carla Ivane Ganz Vogel, Brandon Greene, Johannes Hebebrand, Anke Hinney

**Affiliations:** 1 Institute of Medical Informatics, Biometry and Epidemiology, University of Duisburg-Essen, Essen, Germany; 2 Institute of Medical Biometry and Epidemiology, Philipps-University of Marburg, Marburg, Germany; 3 Institute of Experimental Paediatric Endocrinology, Charité Universitätsmedizin Berlin, Berlin, Germany; 4 Department of Child and Adolescent Psychiatry and Psychotherapy, University of Duisburg-Essen, Essen, Germany; Institute of Preventive Medicine, Denmark

## Abstract

**Background:**

Independent genome-wide association studies (GWAS) showed an obesogenic effect of two single nucleotide polymorphisms (SNP; rs12970134 and rs17782313) more than 150 kb downstream of the melanocortin 4 receptor gene (*MC4R*). It is unclear if the SNPs directly influence MC4R function or expression, or if the SNPs are on a haplotype that predisposes to obesity or includes functionally relevant genetic variation (synthetic association). As both exist, functionally relevant mutations and polymorphisms in the *MC4R* coding region and a robust association downstream of the gene, *MC4R* is an ideal model to explore synthetic association.

**Methodology/Principal Findings:**

We analyzed a genomic region (364.9 kb) encompassing the *MC4R* in GWAS data of 424 obesity trios (extremely obese child/adolescent and both parents). SNP rs12970134 showed the lowest p-value (p = 0.004; relative risk for the obesity effect allele: 1.37); conditional analyses on this SNP revealed that 7 of 78 analyzed SNPs provided independent signals (p≤0.05). These 8 SNPs were used to derive two-marker haplotypes. The three best (according to p-value) haplotype combinations were chosen for confirmation in 363 independent obesity trios. The confirmed obesity effect haplotype includes SNPs 3′ and 5′ of the *MC4R.* Including *MC4R* coding variants in a joint model had almost no impact on the effect size estimators expected under synthetic association.

**Conclusions/Significance:**

A haplotype reaching from a region 5′ of the *MC4R* to a region at least 150 kb from the 3′ end of the gene showed a stronger association to obesity than single SNPs. Synthetic association analyses revealed that *MC4R* coding variants had almost no impact on the association signal. Carriers of the haplotype should be enriched for relevant mutations outside the *MC4R* coding region and could thus be used for re-sequencing approaches. Our data also underscore the problems underlying the identification of relevant mutations depicted by GWAS derived SNPs.

## Introduction

Alleles of common single nucleotide polymorphisms (SNPs) rs12970134 and rs17782313 located downstream of the *MC4R* (154 kb and 188 kb, respectively) were shown to be associated with obesity and related traits [1], [2].

SNP rs17782313 had been identified in a large-scale international cooperation encompassing more than 90,000 individuals [1]. Initially, GWAS data obtained in seven studies (16,876 Europeans) had been meta-analyzed. The second strongest association signal mapped 188 kb downstream of the *MC4R*. This location of rs17782313 indicates that its relevance for weight regulation might be mediated via effects on *MC4R* expression. The association result was confirmed in independent samples of a total of 60,352 adults and 5,988 children. Amongst the adults, each copy of the rs17782313 effect (C) allele was associated with a change in BMI of ∼0.22 kg/m^2^. A copy of the allele resulted in an odds ratio of 1.08 and 1.12 for overweight and obesity, respectively [1].

This small effect size was confirmed by three subsequent GWAS meta-analyses for human obesity genes [3]–[5]. All of these GWAS picked up rs17782313 as a polygenic variant for obesity; a total of more than 150,000 individuals was analyzed [2], [4]–[7].

Additionally, a GWAS for insulin resistance and related phenotypes in 2,684 Indian Asians (including subsequent analyses in 11,955 individuals of Indian Asian or European ancestry) detected an association of rs12970134 located 154 kb downstream of *MC4R* with waist circumference (p = 1.7×10^−9^). The effect was also present in normal weight individuals. Hence, it was suggested that the association of rs12970134 with insulin resistance is partially independent of obesity [2]. Association of rs12970134 with BMI was also found in the whole study group (N = 11,955; p = 6.4×10^−5^; 0.25 kg/m^2^, 95% CI 0.13–0.38 kg/m^2^); in the 4,561 Europeans alone the effect size was similar (0.28 kg/m^2^, 95% CI 0.05–0.51 kg/m^2^).

Currently, it is unclear if and how the association signals of the two common non-coding GWAS-derived SNPs (rs17782313 and rs12970134, with pairwise linkage disequilibrium of D′ = 0.92 and r^2^ = 0.75 according to Ensembl version 57; CEU; Genome Assembly GRCh37) 154 and 188 kb of the 3′ end of the *MC4R* relate to the receptor gene.

On the other hand, approximately 130 mutations have been detected in the human *MC4R* gene; those leading to a reduced function result in a dominantly inherited form of obesity [8]. 2–6% of extremely obese children and adolescents harbour such mutations. Most of these lead to a total or partial loss of function as shown by *in vitro* assays [9]. Quantitative trait analyses in pedigrees revealed that individuals with these mutations had a significantly higher current BMI (4.5 and 9.5 kg/m^2^ in males and females, respectively) than their relatives without the mutations [10]. Phenotypical effects of *MC4R* mutations other than obesity have been shown to encompass hyperinsulinemia, elevated growth rates and higher bone density [11].

In addition to these rare mutations, the minor alleles of two *MC4R* non-synonymous polymorphisms (Val103Ile, Ile251Leu) have convincingly been shown to be protective against obesity [12]–[16]. Heterozygotes for the 103Ile variant of Val103Ile were found in 2–9% of subjects in different populations. An effect size estimate of −0.48 kg/m^2^ for Ile103 carriers was calculated [12] and subsequently confirmed in an extended single large epidemiological study of approximately 8,000 individuals [13]. Additional meta-analyses encompassing a total of 53,343 individuals again confirmed the initial finding [15], [16]. In a GWAS with a focus on early-onset extreme obesity, candidate genes for obesity had specifically been tested. The negative association with obesity for the 103Ile variant of Val103Ile was again supported (p = 4.2×10^−4^ [5]). In the GWAS detecting the association of obesity with the 3′ *MC4R*-SNP [1], the respective finding could not be explained by the two non-synonymous polymorphisms (Val103Ile, Ile251Leu). This conclusion was based on both imputed data with a high confidence score for Val103Ile (posterior probability >0.99 using IMPUTE) and on directly genotyped data for both polymorphisms on a subsample of the study group (Val103Ile, n = 5,516 and Ile251Leu: n = 5,039) [1]. Functional studies showed that the MC4R-103Ile reveals a modest, but significant decrease in antagonist potency [17]. The effect of potent endogenous pro-opiomelanocortin-derived agonists at the MC4R seemed to be increased for MC4R-103Ile [17]. Hence, both the lower antagonistic and the increased agonistic potencies are compatible with an elevated MC4R function, which could explain the weight-reducing effect of the variant.

Finally, a consistent negative association of the Leu251 variant with body weight (minor allele frequency approx. 1 percent) of the second non-synonymous coding polymorphism (Ile251Leu; rs52820871) was detected in about 17,000 individuals of European origin. The effect was shown for extremely obese children and adults (odds ratios ranging from 0.25 to 0.76) and within population-based samples. A meta-analysis supported the evidence of the obesity-protective effect of MC4R Leu251 (odds ratio = 0.52) [14]. An increased basal activity was described for the Leu251 variant [17].

We deem the *MC4R* coding region and the distant SNPs as an excellent example for the recently proposed synthetic association [18], [19]. Accordingly, GWAS signals of common non-functional SNPs outside of coding regions may be the result of a combination of rare coding/functional variants with stronger effects given that these rare variants arose on a haplotype which is tagged by the common SNP. Here we investigate synthetic association by analyzing if common genetic variation (SNPs and haplotypes thereof) genotyped in the non-coding genomic region of the *MC4R* locus is related to coding (functional) variants of the *MC4R*. In the first step, we analyzed the genomic region covering both 3′ SNPs (rs17782313 and rs12970134), the *MC4R* and SNPs in its 5′ flanking region to extract all evidence for common variation related to obesity at the *MC4R* locus. Transmission disequilibrium was analyzed in GWAS data (78 SNPs) of 424 obesity trios. Conditioning on the strongest single-marker signal in our sample, we screened the remaining 77 SNPs for independent (secondary signals with a p-value≤0.05 in the conditional test) signals. Subsequently, we determined the transmission patterns of all possible two-marker haplotypes of the primary and the independent signals. We searched for over-transmission of ‘obesity haplotypes’ to determine if the distant non-coding 3′ SNPs (rs17782313 and rs12970134) belong to haplotypes which extend into the *MC4R* locus. In the second step, we incorporated information on mutations and polymorphisms within the *MC4R* coding region to analyze if synthetic association explains the GWAS signals of the distant SNPs.

## Results

### Extended family-based haplotype analyses of common variants

**Figure 1:**
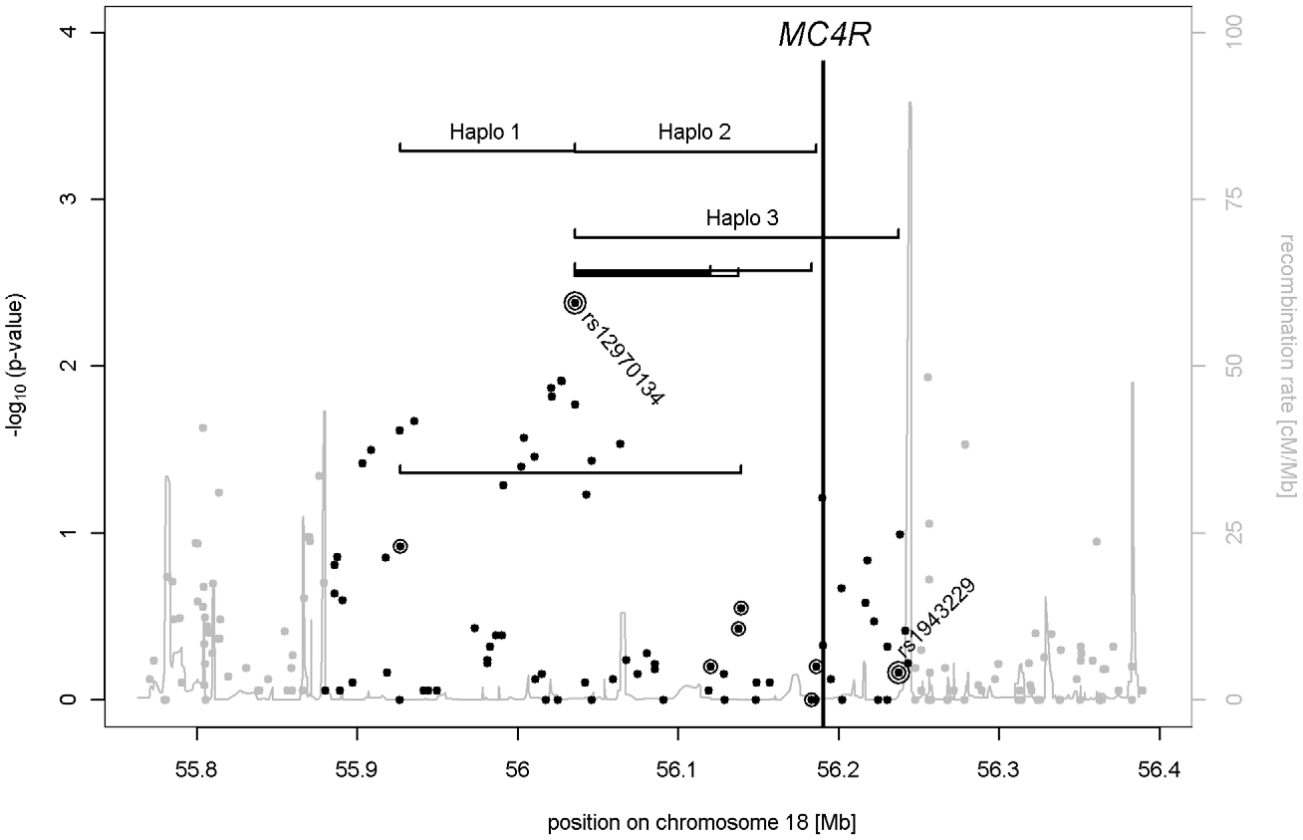
Genomic region encompassing the melanocortin 4 receptor gene (*MC4R*; location indicated by the vertical bar). Displayed are results of transmission disequilibrium tests of single markers and selected two-marker haplotypes in the detection sample of 424 obesity trios (two-sided exact p-values). The black dots indicate single marker TDT results for all 78 SNPs whereas the dots surrounded by a circle highlight the 8 SNPs used for two-marker haplotype construction (selected based on conditional analyses). The region covered by each two-marker haplotype combination is displayed as a line (only those haplotypes with a p-value≤0.1 and whose p-value was below the p-value of each single-marker test are displayed). Surrounding double circles indicate those SNPs (single-marker TDT) of the best supported haplotype result; Haplo 3 spans *MC4R*. In addition, grey lines indicate recombination rates according to HAPMAP CEU.

We analyzed 78 SNPs in the genomic region comprising the *MC4R* gene and reaching from the recombination hotspot at 55,879.013 bps (recombination rate: 43.3 cM/Mb), 310.5 kb 3′ of the *MC4R*, to the recombination hotspot at 56,243,921 bps (recombination rate: 89.5 cM/Mb) 52.9 kb 5′ of the *MC4R* coding region. Analyses were performed in 424 extremely obese German children and adolescents and both of their parents (Affymetrix Genome-Wide Human SNP Array 6.0). The strongest single-marker association signal (according to nominal p-values in the genomic region) was observed for rs12970134 (two-sided exact p = 0.004, relative risk (RR) = 1.37, 95% confidence interval (CI): 1.11–1.69) which had previously been reported by Chambers et al. [2]. SNP rs12970134 was in moderate to strong linkage disequilibrium (LD) to the other previously reported GWAS SNP rs17782313 [1] with D′ = 0.931 and r^2^ = 0.770 (thus, our data were similar to the Ensembl 57 data base entry). A search for secondary signals when conditioning on the strongest signal (rs12970134) revealed that 7 additional SNPs provided independent signals (p≤0.05). Expectedly, rs17782313 did not provide an independent contribution compared to rs12970134 due to the aforementioned LD. The total of 8 SNPs with independent contributions was used to derive the three best two-marker haplotype combinations according to their p-value in the haplotype transmission disequilibrium test (Figure 1).

For replication, all 4 SNPs (rs17700028, rs12970134, rs1943226 and rs1943229) involved in the three best haplotype combinations (p≤0.05 after Bonferroni correction for the 28 haplotypic tests performed) were genotyped in 363 independent obesity trios (Table 1). For one haplotype combination, Haplo 1 (rs17700028 and rs12970134), the haplotype TDT was nominally significant (p≤0.05) in the detection as well as in the replication sample. However, the corresponding risk haplotype [G; A] of Haplo 1 is rare (estimated frequency in the joint sample of 787 parents≈0.2%) and does not provide evidence for an association in the joint sample (haplotype RR (hRR) = 3.24 with 95% CI: 0.36–29.12, p-value = 0.29). Analysis of the full set of 787 obesity trios showed a higher hRR for the risk haplotype combination Haplo 3 than for any other single marker or haplotype combination (Tables 1 and 2). Interestingly, Haplo 3 covers the *MC4R* (Figure 1). In more detail, the combination of the risk alleles of the SNPs included in Haplo 3 resulted in a hRR for [A; G] = 1.61, (95% CI: 1.30–2.00) for obesity, which was descriptively stronger than the relative risks for each of the single markers effect alleles (rs12970134; A is obesity effect allele: RR = 1.33, 95% CI: 1.14–1.55; rs1943229; G is obesity effect allele: RR = 1.03, 95% CI: 0.86–1.23). Note that the single-marker association signal for rs1943229 resulted in p-value = 0.86 in the joint sample (Table 2).

**Table 1:** Global transmission disequilibrium tests (HAP-TDT) of all three best two-marker haplotype combinations (according to p-values) in the genomic region covering the melanocortin 4 receptor gene (*MC4R*) in 424 obesity trios of the detection sample, the confirmation sample of 363 obesity trios and in the joint sample of 787 obesity trios.

	1^st^ SNP of two-marker haplotype	chrom.18 physical position in base pairs^a^	2^nd^ SNP of two-marker haplotype	chrom.18 physical position in base pairs^a^	detection sample (424 obesity trios) p-value^b^	confirmation sample (363 obesity trios) p-value^b^	joint sample (787 obesity trios) p-value^b^	joint sample (787 obesity trios) hRR^c^ for obesity risk haplotype [95% CI]
**Haplo 1**	rs17700028	55,926,664	rs12970134	56,035,730	5.2×10^−4^	0.039	5.7×10^−4^	3.24 [0.36–29.12]
**Haplo 2**	rs12970134	56,035,730	rs1943226	56,186,184	5.2×10^−4^	0.093	6.0×10^−5^	1.48 [1.23–1.78]
**Haplo 3**	rs12970134	56,035,730	rs1943229	56,237,438	0.001	0.074	1.7×10^−4^	1.61 [1.30–2.00]

a base pair position on chromosome 18 according to http://www.ncbi.nlm.nih.gov (hg18);

b permutation-based p-value based on 10^6^ simulations;

c haplotypic relative risk (hRR) relative to the most frequent haplotype for the haplotype comprising the two obesity risk alleles of the 1^st^ and 2^nd^ SNP with 95% confidence intervals (95% CI).

**Table 2:** Single-marker transmission disequilibrium tests in the detection sample (424 obesity trios), the confirmation sample (363 obesity trios) and in the joint sample (787 obesity trios).

SNP	chrom.18 physical position in base pairs^a^	obesity effect /other allele	sample	EAF^b^ in parents [%]	obesity allele transmissions	other allele transmissions	relative risk for effect allele (95% CI^c^)	two-sided exact p-value TDT^d^
rs17700028	55,926,664	[**G**; A]	detection	8.08	75	56	1.34 (0.95–1.89)	0.120
		[**A**; G]	confirmation	90.48	73	50	1.47 (1.02–2.12)	0.047
		[**A**; G]	joint	91.26	129	125	1.03 (0.81–1.32)	1.000
rs12970134	56,035,730	[**A**; G]	detection	30.54	205	150	1.37 (1.11–1.69)	0.004
		[**A**; G]	confirmation	27.55	169	131	1.29 (1.03–1.62)	0.045
		[**A**; G]	joint	29.15	374	281	1.33 (1.14–1.55)	2.5×10^−4^
rs1943226	56,186,184	[**T**; G]	detection	89.51	82	77	1.06 (0.69–1.28)	0.750
		[**G**; T]	confirmation	10.34	65	62	1.05 (0.74–1.49)	0.630
		[**T**; G]	joint	89.58	144	142	1.01 (0.81–1.28)	0.860
rs1943229	56,035,730	[**G**; T]	detection	79.91	141	136	1.04 (0.82–1.31)	0.680
		[**G**; T]	confirmation	80.72	112	110	1.02 (0.78–1.32)	1.000
		[**G**; T]	joint	80.28	253	246	1.03 (0.86–1.23)	0.860

a base pair position on chromosome 18 according to http://www.ncbi.nlm.nih.gov (hg18);

b obesity effect allele frequency;

c confidence interval;

d p-values were calculated using PLINKs′ adaptive permutation procedure with 10^6^ permutations.

Displayed are the SNPs included in the three best haplotypes (Table 1) for the explored genomic region surrounding the melanocortin 4 receptor gene (MC4R, location: chromosome 18: 55,879,013–56,243,921 bps). The obesity-predisposing allele (obesity effect allele) within each sample is highlighted in bold.

### Synthetic association – assessment of the relationship of MC4R coding variants to common obesity-associated SNPs outside of the MC4R coding region

As both, common distant SNPs and functionally relevant low frequency genetic variation within *MC4R*, are associated with obesity, the *MC4R* might be an example for ‘synthetic association’. Accordingly, we evaluated the impact of *MC4R* coding variants on the association signals of the distant SNPs or their respective two-marker haplotype. First, we explored the impact of the non-synonymous polymorphisms Val103Ile and Ile251Leu. Secondly, we assessed the impact of mutations in the *MC4R* coding region that lead to a reduced receptor function.

The obesity effect alleles of the non-synonymous polymorphisms Val103Ile and Ile251Leu (the wild-type alleles G and T) were frequently detected on the common obesity haplotype comprising the obesity effect alleles at rs12970134 and rs1943229 (Table 3; left panel). As expected, pairwise LD measures between the two non-synonymous *MC4R* polymorphisms and the two SNPs of the obesity haplotype are larger for D′ (range 0.475–1), whereas the respective r^2^ values were low (range 0.001–0.007) due to the low allele frequencies of these polymorphisms.

**Table 3:** *MC4R* coding variants and their relationship to haplotype frequencies and transmission ratios of the two-marker haplotype comprising the SNPs rs12970134 (3′end) and rs1943229 (5′end) in the *MC4R* region.

*MC4R* coding region	haplotype [rs12970134; rs1943229] (obesity effect allele)^a^	non-synonymous *MC4R* polymorphisms (Val103Ile and Ile251Leu) whose major (wild-type) alleles are associated with obesity (787 obesity trios)^b^					functionally relevant obesity *MC4R* mutations (525 obesity trios)^c^				
		expected frequency in parents under indepen-dence [%]	estimated frequency in parents [%]	trans-mitted^d^	non-trans-mitted^d^	trans-missionratio^e^	expected frequency in parents under indepen-dence [%]	estimated frequency in parents [%]	trans-mitted^d^	non-trans-mitted^d^	trans-missionratio^e^
*MC4R* wild-type	[G; **G**]	61.3	62.2	346.0	412.1	0.84	62.5	64.0	224.2	286.5	0.78
	[**A**; **G**]	14.9	15.6	246.0	158.0	1.56	15.6	15.5	168.6	103.9	1.62
	[**A**; T]	12.7	13.4	177.8	172.8	1.03	13.1	13.2	118.2	122.1	0.97
	[G; T]	6.0	3.3	89.2	98.2	0.91	6.5	6.3	61.0	70.5	0.87
presence of any *MC4R* variation^a^	[G; **G**]	3.2	2.4	27.82	43.76	0.64	1.5	0.5	7.6	5.6	1.36
	[**A**; **G**]	0.8	0.1	1.18	2.22	0.53	0.4	0.2	5.6	0	-
	[**A**; T]	0.7	0	-	-	-	0.3	0.2	3.7	0	-
	[G; T]	0.3	0.03	0.002	1.02	0.002	0.2	0.1	1.1	1.4	0.8

a note that the minor alleles of the two non-synonymous polymorphisms (Val103Ile and Ile251Leu; left panel) are negatively associated with obesity whereas the functionally relevant mutations (right) are all obesity-related;

b here, all 787 obesity trios were incorporated in the haplotype estimation;

c here, a subset of 525 obesity trios for which information on functionally relevant obesity *MC4R* mutations was available and incorporated in the haplotype estimation;

d decimals in transmitted and non-transmitted haplotypes result from haplotype estimations;

e transmission ratios were obtained from FAMHAP (version 18).

We observed a slightly stronger transmission disequilibrium of the obesity haplotype [A; G] of Haplo 3 (transmission-ratio = 1.56; Table 3) if it included the wild-type, ‘obesogenic’ alleles of the two non-synonymous *MC4R* polymorphisms as compared to the situation of an un-stratified assessment of omitting the two polymorphisms (transmission-ratio = 1.55; Table 4). Accordingly, the obesity alleles of the common obesity-associated SNPs outside of the *MC4R* coding region (rs12970134 and rs1943229) were less frequently transmitted to obese offspring in the presence of the weight-lowering variants of the two *MC4R* polymorphisms (Val103Ile and Ile251Leu; Table 3; left panel) as indicated by transmission-ratios below 1. Subsequently, we removed the heterozygous carriers (total n = 108) at Val103Ile (n = 85) and Ile251Leu (n = 23) from the full sample to explore their impact on the TDT findings for the two distant SNPs (rs12970134, rs1943229). While both the obesity effects for the one distant SNP rs12970134 and the risk haplotype of Haplo 3 associated to obesity (TDT p-value = 0.02 after Bonferroni correction for the 78 tests performed) increased slightly (A allele: RR 1.33 to 1.35; [A; G] allele: hRR 1.61 to 1.64) the point estimate for the other distant SNP rs1943229 did not change (uncorrected TDT p-value = 0.86; G allele: RR 1.03). Similarly, joint modelling of the effects of the two distant SNPs and the non-synonymous *MC4R* polymorphisms collapsed into one covariate [20] resulted in hardly any change of the obesity effects of the two distant SNPs (Table 5; left panel; rs12970134, A allele: RR 1.45 to 1.43; rs1943229, G allele: RR 1.23 to 1.24), while the effect for the collapsed wild-type alleles of the two non-synonymous *MC4R* polymorphisms was slightly reduced (RR 1.66 to 1.54 [20]).

**Table 4:** Analyses for the two-marker haplotype comprising the SNPs rs12970134 (3′end) and rs1943229 (5′end) in the *MC4R* region.

haplotype [rs12970134;rs1943229] (obesity effect allele)	sample	frequency in parents [%]	transmitted^a^	non-transmitted^a^	transmission ratio^b^	hRR^c^ (95% CI^d^)	nominal two-sided p-value (hRR^c^)
	detection	62.8	173.6	229.0	0.76	ref	–
[G; **G**]	confirmation	66.5	154.9	181.4	0.85	ref	–
	joint	64.7	328.6	410.7	0.80	ref	–
	detection	17.1	146.4	88.0	1.66	1.70 (1.28–2.25)	2.8×10^−4^
**[A; G]**	confirmation	14.2	99.1	70.6	1.40	1.49 (1.08–2.06)	0.016
	joint	15.7	245.4	158.3	1.55	1.61 (1.30–2.00)	1.3×10^−5^
	detection	13.3	94.6	99.0	0.96	1.02 (0.74–1.42)	0.89
[**A**: T]	confirmation	13.4	82.9	73.4	1.13	1.04 (0.74–1.46)	0.82
	joint	13.4	177.6	172.7	1.03	1.03 (0.82–1.30)	0.81
	detection	6.7	52.4	51.0	1.03	1.18 (0.78–1.80)	0.43
[G; T]	confirmation	5.9	37.1	48.6	0.76	0.84 (0.76–1.86)	0.45
	joint	6.3	89.4	99.3	0.90	1.01 (0.74–1.37)	0.95

a decimals in transmitted and non-transmitted haplotypes result from haplotype estimation;

b transmission ratios were obtained from FAMHAP (version 18);

c haplotypic relative risks calculated by conditional logistic regression using the R-package “DGCgenetics” (additive haplotypic model);

d confidence interval.

**Table 5:** Conditional logistic regression analysis including effects of the two-marker haplotype SNPs (rs12970134, rs1943229), the two coding polymorphisms (weight lowering effect: Val103Ile, Ile251Leu) and any other functionally relevant obesity *MC4R* mutations.

regression model^a^		non-synonymous *MC4R* polymorphisms (Val103Ile and Ile251Leu) whose major (wild-type) alleles are associated with obesity^b^ (787 obesity trios)				functionally relevant obesity *MC4R* mutations^c^ (525 obesity trios)			
		obesity effect allele	RR for effect allele	95% CI for RR for effect allele	p-value	obesity effect allele	RR for effect allele	95% CI for RR for effect allele	p-value
model 1	rs12970134	**A**	1.45	1.21–1.73	4.5×10^−5^	**A**	1.55	1.24–1.92	8.9×10^−5^
	rs1943229	**G**	1.23	1.01–1.52	0.042	**G**	1.32	1.04–1.68	0.024
model 2	*MC4R* coding region variant	collapsed Val103Ile and Ile251Leu **wild-type allele**	1.66	1.04–2.63	0.032	collapsed Val103Ile and Ile251Leu **wild-type allele**	1.48	0.85–2.57	0.170
						collapsed functionally relevant obesity mutations^c^ **mutated allele**	2.57	1.07–6.15	0.034
model 3	rs12970134	**A**	1.43	1.20–1.71	7.9×10^−5^	**A**	1.52	1.22–1.90	1.8×10^−4^
	rs1943229	**G**	1.24	1.01–1.51	0.042	**G**	1.30	1.02–1.65	0.037
	*MC4R* coding region variant	collapsed Val103Ile and Ile251Leu **wild-type allele**	1.54	0.96–2.48	0.073	collapsed Val103Ile and Ile251Leu **wild-type allele**	1.37	0.77–2.45	0.290
						collapsed functionally relevant obesity mutations^c^ **mutated allele**	2.91	1.05–8.09	0.041

a model 1 included rs12970134, rs1943229 whereas model 2 included either the non-synonymous MC4R polymorphisms (Val103Ile and Ile251Leu) (left and right panel) or the functionally relevant obesity MC4R mutations (right panel) as one explanatory variables; in model 3, both rs12970134, rs1943229 and the coding variants were included;

b wild-type alleles at the non-synonymous MC4R polymorphisms (Val103Ile and Ile251Leu) were combined into one covariable; c mutation alleles Ser30Phe, [Tyr35Stop; 110A>T], Pro78Leu, Ser94Arg, Thr112Met, Ile121Thr, Ser127Leu, Arg165Trp, Ala175Thr, Gly181Asp, Met200Val, Ala244Glu, L211fsX216, Ile317Thr were combined into one covariable; the 525 obesity trios are a subset of all 787 trios for which information on functionally relevant obesity MC4R mutations were available.

In a subgroup (n = 525, see [12], [21]) of the 787 trios we also analyzed if mutations in the *MC4R* coding region that lead to a reduced receptor function can in part explain the effect observed for the common obesity-associated SNPs outside of the *MC4R* coding region. We had previously screened the *MC4R* coding region for mutations (dHPLC [21]). We observed heterozygous carriers of the following *MC4R* mutations that lead to a reduced receptor function as shown by at least one functional assay: Ser30Phe [9], [Tyr35Stop; 110A>T] [9], Pro78Leu [22], Ser94Arg [21], [23], Thr112Met [24], Ile121Thr [21], [23], Ser127Leu [9], [21], Arg165Trp [24], [25], Ala175Thr [26], Gly181Asp [21], [23], [27], Ala244Glu [21], deletion of 4 base pairs at codon 211 (L211fsX216 [21], [26]; Ile317Thr [24]. For one mutation (Met200Val), two functional assays (cAMP response and cell surface expression) revealed a function similar to the wild-type MC4R [9], [28]. However, for some of the other mutations, there was only one amongst a variety of assays that showed a deviation from the wild-type receptor (e.g. Thr112Met; wild-type function in [9], [21], [23]; only Nijenhuis et al. [24] described a reduced receptor function). Hence, we decided to also rate Met200Val as a functionally relevant mutation; rating as a wild-type receptor did not alter the results (data not shown).

Again, removal of the mutation carriers from the analysis did not alter the results for the distant SNPs or the two-marker haplotype strongly (rs12970134-A: RR 1.38 to 1.34; rs1943229-G: RR 1.05 to 1.03; hRR for [A; G] of Haplo 3: 1.82 to 1.70). For the more distant marker rs12970134 and for the obesity effect haplotype [A; G] of Haplo 3, the effect was slightly reduced in comparison to the model of not removing the mutation carriers. For rs1943229 located closer to the coding region of the *MC4R* the association signal remained nearly the same upon removal of the mutation carriers. Similar observations were made for the joint modelling of the distant SNPs, the non-synonymous *MC4R* polymorphisms and the mutations (Table 5; right panel). For the joint modelling, we collapsed the wild-type alleles of the non-synonymous *MC4R* polymorphisms into one covariate and all mutations into another [20] as each single *MC4R* mutation is almost a private event. Here, in contrast to the observation for the non-synonymous *MC4R* polymorphisms Val103Ile and Ile251Leu, the combination of all functional mutations (n = 15) resulted in an additional independent obesity-association signal for the mutations (p-values≤0.05 in model 2 analyzing the mutations only and model 3 joint modelling of distant SNPs, the non-synonymous *MC4R* polymorphisms and the mutations; Table 5 right panel) which was even strengthened (RR 2.57 to 2.91) upon inclusion of the polymorphisms.

## Discussion

At the *MC4R* locus, common SNPs are associated with polygenic forms of obesity and variants leading to a reduced MC4R function entail a major gene effect for obesity. Thus this gene locus is ideally suited for the analysis of synthetic association. We performed conditional as well as haplotype analyses for a genomic region approximately 310 kb downstream of the *MC4R* to 53 kb upstream of the *MC4R* coding region (between two recombination hotspots). In contrast to prior analyses [1], we had the advantage of being able to use directly genotyped data from nuclear families. Thus, we did not have to rely on imputed genotypes; estimation of haplotypes is simplified and transmission patterns can be explored.

Haplotype analyses are known to be more powerful than single-marker analyses for the detection of a genomic region that is enriched for phenotype-relevant mutation(s)/causal variant(s). Recently, the analysis of WTCCC (Wellcome Trust Case Control Consortium) data sets of seven complex disorders suggested that haplotype analyses in current GWAS can guide future re-sequencing approaches to identify underlying rare functional variants [29]. Therefore, we analyzed haplotypes flanking the *MC4R*. We observed a stronger association signal upon combining the information of SNPs from the 3′ and 5′ regions than for each single SNP in two independent TDT-based association studies. Whether the constructed obesity haplotype points to an ancestral obesity haplotype or if it reflects the impact of two relatively independent loci in the vicinity of the *MC4R* is beyond the scope of our analyses. However, as we have limited our analysis to the region between the recombination hotspots derived from 30 CEU Hapmap trios [1] 3′ and 5′ to the *MC4R* we attempted to increase the chance to detect an ancestral haplotype.

As we identified a relatively common risk haplotype (frequency of [A; G] of Haplo 3 equals to 15.7% in the 787 parents) covering the *MC4R* coding region, we also analyzed if the associations of obesity to the distal 3′ common SNPs represent an example of synthetic association [18], [19]. Synthetic association implies that GWAS signals of common non-functional SNPs outside of coding regions may be the result of a combination of rare coding/functional variants with stronger effects. This is in contrast to the more widespread idea that association signals of common non-functional SNPs outside of coding regions point to a common causal variant.

To further explore this idea, we analyzed the influence of coding variants (two coding polymorphisms and several functionally relevant rare mutations) in *MC4R* on the transmission of the obesity effect haplotype as well as on the obesity effect alleles of the two SNPs at the 3′ and 5′ ends forming the obesity effect haplotype. Both the removal of individuals with *MC4R* coding variants and the inclusion of the information on *MC4R* coding variant status in a regression model had basically no impact on the estimators of the obesity effect alleles of two SNPs at the 3′ and 5′ ends of the *MC4R*, as well as for the obesity effect haplotype. This observation did not support the model of synthetic association [18] for the *MC4R* coding region. Thus, we conclude that the investigated genetic variation within the coding region cannot explain the obesity association effect of the 3′ SNP rs12970134 or that of its haplotype combinations with rs12970134 (Haplo 3) observed in our samples. However, we cannot exclude that other functional variants [30]–[32] outside of the coding region of the *MC4R* contribute to the GWAS obesity association signal. Moreover, we cannot exclude that other less frequent non-coding variants not properly tagged by the current GWAS technology may be correlated to the *MC4R* coding variants. Finally, our analyses are based on a relatively small sample size, which results in low power to detect potentially small synthetic association effects.

In sum, we detected a haplotype covering the *MC4R* coding region; or at least a secondary independent signal 5′ of the *MC4R* which is associated with extreme obesity. Recently, a meta-analysis comprising GWAS data of 123,865 individuals of European ancestry followed by confirmatory analyses in up to 125,931 independent individuals also described a secondary independent signal at the *MC4R* locus at a position similar to the one we detected [33].

We observed a stronger association signal for an obesity effect haplotype as compared to single-marker signals in both our detection and our confirmatory samples. Accounting for genetic variation in the *MC4R* coding region following the idea of synthetic association had hardly any impact on the association signal in our sample. Consequently, the genomic region of Haplo 3 (201,708 base pairs) could be a focus of a deep sequencing approach aiming at the detection of additional obesity mutations/polymorphisms outside of the *MC4R* coding region. We aim at re-sequencing this region in the extremely obese individuals harbouring the risk haplotype ([A; G] of Haplo 3); subsequently we will analyze [34] newly identified potentially causal rare variants in independent well-powered case-control samples.

## Materials and Methods

### Study group

A total of 787 obesity trios comprising an extremely obese child or adolescent (index patients, see [35]) and both of their biological parents (for details see Table 6) was analyzed. Written informed consent was given by all participants and in case of minors by their parents. The study was approved by the Ethics Committees of the Universities of Marburg and Essen and conducted in accordance with the guidelines of *The Declaration of Helsinki*.

**Table 6:** Description of the investigated two independent German family-based data sets (detection and confirmation sample).

Study group		status	n total (female)	age [years] (mean; SD)	BMI [m/kg^2^] (mean; SD)	BMI Z-score (mean; SD)^a^
detection: obesity trio genome-wide association study	extremely obese children and adolescents	index patients	424 (224)	13.20 (2.80)	31.93 (5.39)	4.22 (1.84)
	parents of the obese children and adolescents	parents	848 (424)	42.32 (6.07)	30.40 (6.44)	1.66 (1.85)
confirmation: obesity trios genotyped for four SNPs	extremely obese children and adolescents	index patients	363 (209)	13.38 (3.40)	32.46 (6.45)	4.35 (2.15)
	parents of the obese children and adolescents	parents	726 (363)	40.62 (5.92)	30.42 (6.15)	1.72 (1.81)

a [33].

### Genotyping

Genotyping in 424 obesity trios was performed on the Genome-Wide Human SNP Array 6.0 (http://www.affymetrix.com) by ATLAS Biolabs GmbH (Berlin, Germany). Birdseed V-2 algorithm was applied for calling. For genotyping of rs12970134 and rs1943229 we used available TaqMan assays (C___3058722_10 and C__11962333_10, Applied Biosystems, Germany). The variants rs17700028 and rs1943226 were genotyped using ARMS-PCR [36], primers are available upon request. The non-synonymous *MC4R* polymorphisms Val103Ile (rs2229616 – also available on the Genome-Wide Human SNP Array 6.0) and Ile251Leu (rs52820871) were genotyped as described previously [22]. To validate the genotypes, allele determination was made independently by at least two experienced individuals. Discrepancies were solved unambiguously either by reaching consensus or by repeating.

### Mutation screen

For the functionally relevant obesity *MC4R* mutations, we used the previously published mutation screen data (525 trios) [21].

### Statistical analyses

For the GWAS data on 424 obesity trios, SNPs with a call rate <95%, departure from Hardy-Weinberg equilibrium in the parents (two-sided exact test <0.001), or with minor allele frequency below 1 percent in the parents were excluded from the analysis (20 SNPs in the ±200 kb region as described below). In addition, all SNPs were set to “missing” in case of Mendelian inconsistent calls within a family. From the genome-wide data set, 78 SNPs were selected that met the quality control criteria and which were within a genomic region defined as 200 kb 3′ of the most distant previously reported GWAS SNP (rs17782313) [1] to 200 kb 5′ of the *MC4R* coding region. On this marker set, we performed single-marker transmission disequilibrium tests (TDTs) [37] using PLINK v1.07. Relative Risks were estimated by use of conditional logistic regression. Subsequently, we aimed to screen for a set of SNPs which most likely contributed secondary independent association signals. We performed conditional analyses on the SNP (rs12970134) with the strongest primary signal using FAMHAP (version 18) [38]. This test is based on the idea that transmission of haplotypes of closely linked SNPs involving rs12970134 would not depend on allelic status at another SNP, if rs12970134 is the only causal variant. For screening purposes, we selected those SNPs with a permutation-based p-value below 0.05 in the conditional test. Afterwards, we performed family-based association tests of all two-marker haplotypes for the set of selected SNPs (primary and secondary signals) using the weighted haplotype test as provided in FAMHAP (HAP-TDT) [38]. Again, permutation-based p-values were derived. In addition, to overcome the problem of haplotype phasing in the possible presence of some recombinations, we estimated effect sizes such as relative risks with conditional logistic regression under a log-additive mode of inheritance applying the R-package ‘DGCgenetics’ provided by David Clayton. To address the inherent multiplicity and over-fitting problem in the GWAS detection sample, we limited the analyses in the independent confirmation sample (363 obesity case-parents trios) to those models including the two SNPs of Haplo 3 (rs12970134, rs1943229). Using the observed data from the detection data set and simplified adapting it to the bi-allelic case to derive a power estimate for the confirmatory sample using Quanto 1.2.4, we estimated that 363 trios would have a power of 97% to detect an RR effect of RR = 1.7 under a log-additive mode of inheritance (minor allele frequency 15%, α_two-sided_ = 0.05).

Finally, we jointly analyzed all 787 trios. For the exploration of the genomic region in order to support the idea of ‘synthetic association’, we phased our data sets using FAMHAP (version 18) [38]. Additionally, we applied conditional logistic regression analyses as implemented in R v.2.9.0 incorporating the SNPs rs12970134, and rs1943229 as well as the variants in the *MC4R* coding region as independent covariants hierarchically. To enable estimation, we adopted the idea of Li and Leal [20] and collapsed effects of similar variant classes (non-synonymous *MC4R* polymorphisms or functionally relevant obesity *MC4R* mutations). Linkage disequilibrium measurements for the polymorphisms rs12970134, rs1943229, Val103Ile (rs2229616) and Ile251Leu (rs52820871) were based on haplotype phasing incorporating all four variants.
